# Phenotypic and treatment outcome data on SUNCT and SUNA, including a randomised placebo-controlled trial

**DOI:** 10.1177/0333102417739304

**Published:** 2017-11-02

**Authors:** Hsing-Yu Weng, Anna S Cohen, Christoph Schankin, Peter J Goadsby

**Affiliations:** 1 Department of Neurology, Wan Fang Hospital, Taipei Medical University, and Department of Neurology, School of Medicine, College of Medicine, Taipei Medical University, Taipei, Taiwan; 2 University of California, San Francisco, San Francisco, San Francisco CA, USA; 3 Clinical Neurosciences, Royal Free Hospital, London, UK; 4 Department of Neurology, Inselspital, Bern University Hospital, University of Bern, Bern, Switzerland; 5 NIHR-Wellcome Trust King’s Clinical Research Facility, King’s College London, UK

**Keywords:** SUNCT, SUNA, trigeminal autonomic cephalalgia, cranial autonomic symptoms, triggers, lidocaine, lamotrigine, topiramate, gabapentin

## Abstract

**Background:**

Short-lasting unilateral neuralgiform headache attacks with conjunctival injection and tearing (SUNCT) and short-lasting unilateral neuralgiform headache attacks with cranial autonomic symptoms (SUNA) are two rare headache syndromes classified broadly as Trigeminal Autonomic Cephalalgias (TACs).

**Methods:**

Here, 65 SUNCT (37 males) and 37 SUNA (18 males) patients were studied to describe their clinical manifestations and responses to treatment.

**Results:**

Pain was almost always unilateral and side-locked. There were three types of attack: Single stabs, stab groups, and a saw-tooth pattern, with some patients experiencing a mixture of two types. As to cranial autonomic symptoms, SUNA patients mainly had lacrimation (41%) and ptosis (40%). Most cases of the two syndromes had attack triggers, and the most common triggers were touching, chewing, or eating for SUNCT, and chewing/eating and touching for SUNA. More than half of each group had a personal or family history of migraine that resulted in more likely photophobia, phonophobia and persistent pain between attacks. For short-term prevention, both syndromes were highly responsive to intravenous lidocaine by infusion; for long-term prevention, lamotrigine and topiramate were effective for SUNCT, and lamotrigine and gabapentin were efficacious in preventing SUNA attacks. A randomized placebo-controlled cross-over trial of topiramate in SUNCT using an *N-of-1* design demonstrated it to be an effective treatment in line with clinical experience.

**Conclusions:**

SUNCT and SUNA are rare primary headache disorders that are distinct and very often tractable to medical therapy.

## Introduction

Short-lasting unilateral neuralgiform headache attacks with conjunctival injection and tearing (SUNCT) is a rare form of primary headache (1,2). It is clear in tertiary headache practice that many patients do not manifest both conjunctival injection and tearing (3). The current terminology has evolved to respect the initial description and acronym, and acknowledge the underlying physiological principle of co-existent cranial autonomic activation (4). SUNCT syndrome was initially included in the second edition of the International Headache Classification and SUNA in the appendix (5). In the latest version, ICHD-3 beta (6), SUNCT and SUNA are included in the main body.

At least two unresolved issues arise in these syndromes. First, should they be collapsed under an umbrella or left distinct? Given their rarity, substantial series have not been available to explore the phenotypes. Previously, we reported on 43 SUNCT and nine SUNA patients (3); in the following decade, we have seen more patients with these syndromes and sought here to examine whether the syndromes are sufficiently distinct to maintain their separation. Moreover, we had noted in previous work that migraine features in the phenotype appeared to be associated with an underlying migrainous biology (3); we, therefore, wished to test the question as to whether having underlying migrainous biology influenced the phenotypic expression of these syndromes with our expanded cohort. Secondly, while there are treatment guidelines (7), the body of evidence for the guidance is minimal. We have been able to collect substantial treatment response data, and uniquely have conducted a randomized placebo-controlled trial of SUNCT to test whether topiramate is an effective treatment. We therefore set out to provide experience as an evidence base (8) for treatment recommendations.

## Material and methods

### Clinical material

All patients attended outpatient clinics at either the National Hospital for Neurology and Neurosurgery (NHNN), London, UK, between 2002 and 2007; the Headache Center, University of California, San Francisco (UCSF), San Francisco, CA, USA, from 2007 to 2013, or as outpatients at King’s College Hospital, London, UK from 2013 to 2015. Patients were diagnosed as having SUNCT or SUNA as defined by the International Classification of Headache Disorders, Second Edition (ICHD-2) (5) and were consistent with proposed ICHD-3 beta criteria (6). Every patient was seen by at least one of us (PJG). The cohort represents those previously reported (3) whose clinical data were re-reviewed, and additional cases. The study was approved by the NHNN Institute of Neurology Joint Research Ethics Committee (reference 00/N072), by the UCSF Committee for Human Research, and is presented as an audit of practice at King’s College Hospital, London.

In every patient, detailed and standardised history-taking was carried out, including the side, location, frequency, duration, and character of their headaches, types of attacks, accompanying symptoms, triggering factors, personal and family histories, and their responses to medicines or other treatments. The study focuses on lateralization and type of attacks, cranial autonomic symptoms, and treatment outcomes.

#### Clinical data analysis

All data were recorded using Microsoft Excel. All descriptive calculations were performed in Excel. Summary measures are reported for the cohort as n’s with percentages. To test whether the SUNCT and SUNA are different based on phenotype as a surrogate for biology, apart from the cranial autonomic symptom distinction, we used a multinomial logistic model, with a logit link function, and diagnosis as the dependent variable (IBM SPSS Statistics v 22). To test the relationship of background migrainous biology, defined as a personal or family history of migraine, to the presence of a clinical phenotype, we used a binary logistic model, with a logit link function, and the presence of migrainous biology as a binary dependent variable. To test single phenotype questions, a chi squared test was employed. For all analyses, significance was set as *p* < 0.05. The clinical effect from preventives is recorded as patients subjectively reported their effects, and as it was recorded in the documentation.

### Clinical trial

**Figure 1.**
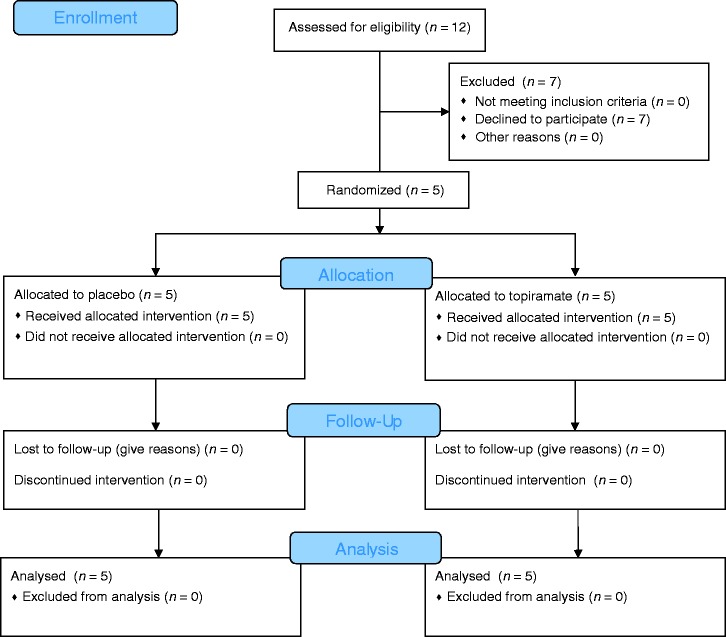
CONSORT flow diagram.

Five male patients (aged 51–72, mean 59 years) with SUNCT were recruited from the Outpatient department at the National Hospital for Neurology and Neurosurgery (NHNN), London, between 2003 and 2004 (Figure 1). They were initially diagnosed with SUNCT using operational criteria later incorporated into ICHD-2 (5). Inclusion criteria were: Diagnosis of SUNCT; willingness to stop any current treatments; and willingness to comply with a diary of attacks as they occurred. Exclusion criteria were: Previous exposure to topiramate; being pregnant or lactating; having a history of renal calculi; and any contraindications to the use of topiramate according to the Summary of Product Characteristics (Medicines and Healthcare products Regulatory Agency- MHRA, UK). The subjects gave their informed consent and were free to withdraw from the study at any time. The study was conducted before trial registration became commonplace (9).

#### Design

The design of the study was a randomised, double-blind, placebo-controlled crossover trial of topiramate. The treatments were topiramate tablets and matching placebo, supplied by Janssen UK; and labelled Treatment 1 and Treatment 2, to be taken in the first and second arm of the study, respectively. The order of active treatment and placebo was randomised by NHNN pharmacy, and each participant was assigned a randomisation number. The code was held by the pharmacy until study completion and database locking.

#### Conduct

Patients were required to withdraw from preventive medications prior to commencement of the study. After an initial washout drug-free period of 10 days, treatment was started at topiramate 12.5 mg at night and increased every five days to a maximum of 50 mg twice daily for 10 days, after which the dose was reduced over the next 10 days, or the same regimen with matched placebo. A 10-day washout drug-free period followed, after which the patients commenced the second arm of the study in the same paradigm. The patients with episodic SUNCT started the 10-day washout period at the start of their bout.

The patients were instructed to keep a diary for the duration of the study, which documented the date, time, severity, and duration of each attack.

#### Clinical trial data analysis

The primary endpoint was the reduction of attack frequency, as measured by the mean daily number of attacks during the 10 days at maximum dose compared to the 10 drug free days pre-treatment, comparing active and placebo treatment arms. A secondary endpoint, ‘attack load’, was calculated as the number of minutes of pain per day for each patient, to take account of longer attacks, such as saw-tooth patterns (3). The results are presented on an *N-of-1* basis. A positive result was declared for the endpoint in each patient, if the outcome was reduced by 50% or more compared to placebo. Given SUNCT attack frequency can vary considerably from week to week, we converted changes to percentages and calculated:

%therapeutic effect = (topiramate change) minus (placebo change).

## Results

### Clinical data

#### Subjects

One hundred and two cases were identified: 65 with SUNCT, and 37 with SUNA. There were 37 male and 28 female SUNCT patients, and 18 male and 19 female SUNA patients. The mean age of onset of SUNCT patients was 46 ± 13 (mean ± SD; range: 13–75) years, and that of SUNA was 45 ± 16 (range 15–92).

#### Laterality of attacks

Of all the SUNCT and SUNA patients, 48 (47%) cases had only left-side attacks, and 52 (51%) cases had attacks exclusively on the right side; 12 (12%) cases had unilateral attacks with side variance; one case had a unilateral attack with nearly equal possibility of left or right side; only one case had experienced bilateral attacks.

#### Cranial autonomic symptoms

By definition, every SUNCT patient had attacks with both an ipsilateral conjunctival injection and lacrimation, while less than half of SUNA patients had either. SUNCT is generally more feature-full in terms of cranial autonomic symptoms than is SUNA. SUNA is dominated by lacrimation, nasal symptoms, and ptosis (Table 1).

**Table 1.** Cranial autonomic features.

Feature	SUNCT n (%)	SUNA n (%)
Conjunctival injection	65 (100)	7 (24)
Lacrimation	65 (100)	14 (48)
Nasal: Blocking or rhinorrhea	44 (67)	12 (36)
Aural fullness	12 (50)	5 (17)
Periorbital oedema	25 (41)	8 (29)
Forehead/facial:		
Sweating	4 (7)	1 (3)
Flushing	11 (18)	5 (17)
Sympathetic:		
Ptosis	31 (51)	11 (38)
Miosis	1 (2)	0

### Comparing the phenotypes

#### Triggering

Among SUNCT patients, one patient had only triggered attacks, with no spontaneous ones. Eight (12%) SUNCT patients had only spontaneous attacks with no triggered ones. All the other patients had both spontaneous and triggered attacks. In SUNA patients, 10 (27%) patients had only spontaneous attacks with no triggers, and the remainder had more triggered attacks than spontaneous ones. Of all SUNCT patients, 56 (86%) cases had pain attacks with a cutaneous trigger. The most common triggers included touch (39 cases, 60%), chewing or eating (35 cases, 54%), the wind (24 cases, 37%) and brushing the teeth (23 cases, 35%). Among SUNA patients, 32 (86%) cases had pain with triggers. The most frequent triggers of SUNA cases were chewing or eating (12 cases) and touch (10 cases).

#### Refractory period

Of SUNCT cases for whom we had data, 1/52 had a refractory period to cutaneous triggering. Of SUNA cases, 4/32 had a refractory period after cutaneous triggering.

#### Types of attacks

Attacks of SUNCT and SUNA take one or more of three forms: Single stabs, a group of stabs, or a saw-tooth pattern of stabs. In this cohort, SUNCT patients had more single stabs, while SUNA had more groups of stabs (χ^2^ = 23.4, *p* < 0.0001). The saw-tooth pattern was comparable in both conditions (Table 2).

**Table 2.** Pattern of attacks.

	SUNCT n (%)	SUNA n (%)
Single stabs	41 (64)	8 (37)*
Groups of stabs	23 (36)	23 (62)*
Saw-tooth pattern	22 (34)	9 (24)
Single stabs + groups of stabs	10 (18)	2 (9)
Single stabs + saw-tooth pattern	5 (9)	1 (5)
Groups of stabs + saw-tooth pattern	1 (2)	1 (5)
Single stabs + groups of stabs + saw-tooth pattern	3 (5)	0

* *p* < 0.0001

### Migrainous background

Both SUNCT and SUNA patients had a background migrainous biology in 57 % of cases for which we had data. In a model examining the pain features, a migrainous background was strongly associated with background pain between attacks (Wald χ^2 ^= 6.5, *p* = 0.01) and worsening of pain during the attack (Wald χ^2 ^= 8.2, *p* = 0.004). In a model examining the canonical migraine features, the presence of nausea was not associated with migrainous biology (Wald χ^2 ^= 1.04, *p* = 0.307), whereas the presence of either photophobia or phonophobia was associated (Wald χ^2 ^= 6.8, *p* = 0.009). Worsening of background pain with movement was seen in 26% (n = 55) of SUNCT cases and in 46% (n = 28) of SUNA cases. Nausea was seen in 27% (n = 64) of SUNCT cases and 29% (n = 35) of SUNA cases. Similarly, for photophobia or phonophobia, 61% (n = 64) of SUNCT and 58% (n = 36) of SUNA cases had one, or both, symptoms.

### Treatment effects

#### Short-term prevention

Sumatriptan was used in 14 SUNCT and nine SUNA patients, and the attacks lessened in only one of each. High-flow oxygen was tried, but neither SUNCT nor SUNA cases were responsive. Placebo-controlled indomethacin injections (10) had no effect on either SUNCT or SUNA. Intravenous lidocaine had a very good effect in reducing attacks of SUNCT (100%) and SUNA (88%), when it could be tolerated. Dihydroergotamine (11) and corticosteroids seem generally unhelpful in both SUNCT and SUNA. Greater occipital nerve (GON) injections (12) were beneficial in 50% (6 of 12 cases) of SUNCT patients, but had no reliable effect in SUNA cases (Table 3).

**Table 3.** Effect of acute treatments of TACs on SUNCT and SUNA.

	SUNCT		SUNA	
	Total n	Effective n	Total n	Effective n
Sumatriptan 6 mg sc	14	1	9	1
Oxygen 12–15 L/min 100%	14	0	7	0
Indomethacin 100 mg imi*	13	0	6	0
Lidocaine (iv)	15	15	9	8
Dihydroergotamine, iv over 5 days**	5	0	5	0
Corticosteroids – oral high dose	20	2	6	0
Greater occipital nerve injection***	12	6	4	3

* Placebo controlled (10); **over five days (11); ***with lidocaine and depomethylprednisolone ipsilateral to pain (12).

#### Long-term prevention

Lamotrigine had a good effect in reducing the frequency or severity of the attacks in 62% of SUNCT and 31% of SUNA patients at doses of about 100 to 600 mg/day (Table 4).

**Table 4.** Effectiveness* of preventive.

	SUNCT		SUNA	
	Total n	Effectiveness n (%)	Total n	Effectiveness n (%)
Lamotrigine	29	18 (62)	16	5 (31)
Topiramate	27	13 (48)	9	1
Gabapentin	29	11 (38)	18	7 (39)
Carbamazepine	43	16 (36)	20	4 (20)
Oxcarbazepine	7	1 (14)	6	0
Pregabalin	7	1 (14)	16	1
Verapamil	16	2 (13)	5	0
Valproate	13	0	4	0
Beta-blocker	7	0	4	0
Tricylic	36	3	17	3

* Effectiveness: Reported to be useful in reducing frequency or severity of attacks by the patient.

Topiramate had a good effect in 48% of SUNCT patients at doses of 50–500 mg/day. However, no SUNA case had a good or better response to topiramate.

Gabapentin was used in 18 SUNA patients at a dose of 1800–2400 mg/day; seven cases had good to moderate improvement (39%). In addition, gabapentin was also beneficial in 11 of 29 SUNCT cases.

Carbamazepine also had a good to moderate effect in 36% of SUNCT patients at doses of 100–1200 mg/day. Carbamazepine was effective in 20% of SUNA cases.

### Brain imaging

There were 55 SUNCT (85%) and 33 SUNA (89%) patients with brain imaging, which were almost all magnetic resonance (MR). No abnormal findings on brain images were identified in 42 SUNCT (76%) and 31 SUNA (94%) cases, and apart from pituitary changes, none were SUNCT or SUNA related. There were four SUNCT patients with pituitary lesions. Two of them had macroadenomas, which resolved after treatment of the tumors. The other two had microadenomas, and one of them became pain-free for eight months after resection of the tumor. Vascular loops were found in the brain MR of five SUNCT (10%) and two SUNA (8%) patients. Among them, three SUNCT and two SUNA cases had vascular loops compressing the trigeminal nerves, one was ipsilateral to the pain, one had bilateral loops but unilateral pain only, and one had a vessel loop at one side but had bilateral pain. One SUNCT and two SUNA cases received microvascular decompression: One SUNA case was pain-free for only three months after the surgery, and the operation had no effect in improving the attacks of the SUNCT and the other SUNA cases.

### Clinical trial

All five patients completed the trial (Figure; Table 5).

**Table 5.** Clinical trial: Demographics and outcomes.

	Patient				
	#1	#2	#3	#4	#5
Age (years)	72	54	60	59	51
Duration of SUNCT attacks	120–1800 s	1 s stabs, 3600 s groups	5 s	1 s stabs, 300 s groups	60 s
Daily frequency of attacks pre-placebo	7	13	140	13	10
% change in frequency on placebo	−100	+52	−15	+22	−10
Daily frequency of attacks pre-topiramate	2	23	138	10	6
% effect – frequency topiramate	+2063	−19	−100	+27	−18
Primary endpoint %gain Topiramate − placebo	+2163	−71	−85	+5	−8
Daily attack load (minutes) pre-placebo	3	5	NA	93	11
% change in attack load on placebo	−100	+178	NA	+192	+45
Daily attack load pre-topiramate	57	33	NA	197	3
% change in attack load on topiramate	+920	−71	NA	−17	+160
Secondary endpoint %effect Topiramate − placebo	+1020	−249	NA	−209	+115

Patient #1 had a good effect whilst on placebo, with complete cessation of his attacks. On topiramate, there was an increase of both attack frequency and attack load. He was classified as a topiramate failure; indeed, it appeared to worsen his problem.

Patient #2 had a reduced frequency of attacks by 71% compared to placebo; he was classified a success. He also had a substantial reduction in attack load compared to the placebo period.

Patient #3 had complete cessation of his attacks on topiramate, and a milder 15% reduction on placebo. He was classified as a success. He did not provide attack length data.

Patient #4 had a 5% difference between placebo and topiramate arms in terms of frequency, and was classified as a topiramate failure. On the secondary measure, there was a greater than 200% reduction in attack load.

Patient #5 had an 8% reduction in frequency on the placebo arm when compared to the topiramate arm. He was classified as a failure. On the secondary endpoint of attack load, he worsened by 115 %.

Overall, on the primary endpoint, two patients had improvement, two patients had no change and one worsened.

#### Clinical trial – adverse events

One patient (#4) reported peripheral paraesthesiae on topiramate. One patient (#5) reported peripheral paraesthesiae on both treatments and also had dull headache attacks whilst on both treatments, which were not related to his SUNCT or migraine, and not recorded as such. He had one episode of diplopia lasting an hour on placebo, and reported indigestion whilst on topiramate.

## Discussion

In this substantial cohort of patients with SUNCT and SUNA, we have been able to explore similarities and differences between the conditions, and explore treatment outcomes. The data suggest sufficient differences in core phenotypes to maintain the current classification. Cranial autonomic symptoms are distributed differently, even allowing for the definitional constraint in SUNCT. Attack pain features show SUNCT being more likely to have single stabs and SUNA more likely to have groups of stabs. In terms of preventive treatments, SUNA is generally less responsive, being most likely to be improved by gabapentin or lamotrigine, while SUNCT is more likely to be improved by lamotrigine and topiramate. Our small randomised placebo-controlled trial of topiramate in SUNCT supports the clinical data and, since SUNA seems much less likely to respond, provides another hint that the conditions have some difference. While the data certainly do not settle the issue of whether SUNCT is on a continuum with SUNA, they do suggest it is reasonable from both a clinical and research perspective to maintain the distinction until more data can be collected.

The outcome of treatments is instructive as to the biology of SUNCT/SUNA. The conditions respond very well, almost invariably, to intravenous lidocaine, if it is tolerated. There is no effect of oxygen, which although open label, was administered in a dose effective for cluster headache (13). Similarly, while cluster headache is very likely to respond to sumatriptan by injection (14), one only in each group responded here. Again corticosteroids, which can certainly be helpful in the short term in many patients with cluster headache (15), were not useful in most patients with SUNCT or SUNA. In contrast to paroxysmal hemicrania, where an indomethacin effect is diagnostic (16), no such effect is seen in SUNCT or SUNA. One outcome shared with all the TACs, indeed with migraine (12), is a useful effect of greater occipital nerve injection (GONi) with local anesthetic and corticosteroid. The distinction between that effect and the lack of effect of high dose corticosteroids alone suggests the GONi has more than an effect of corticosteroid dose.

An interesting issue that has arisen in the literature is the relationship between SUNCT/SUNA and trigeminal neuralgia (17). The distribution of pain seems clearly different: SUNCT/SUNA much more often involves the ophthalmic division of the trigeminal nerve, in more than two-thirds of cases (3), while trigeminal neuralgia only involves this division in 4% of cases (18). Cranial autonomic symptoms are the rule in SUNCT/SUNA, with more than 99% of SUNCT/SUNA we have reviewed having one or more, and all SUNCT patients having at least two, while, depending on what cases are accepted, the rate is considerably less in trigeminal neuralgia (18). Less than 5% of SUNCT/SUNA patients presented in our cohort had a refractory period to re-triggering, while almost all trigeminal neuralgia patients have a refractory period. The pain of trigeminal neuralgia is classically described as short electric-shock like pains, whereas up to one-third of our SUNCT/SUNA cohort had the saw-tooth pattern, which is invariably longer than a less than one second stab. Interestingly, both conditions have background pain in about half of patients (19); this would fit well to a common basis for that pain, i.e. associated migrainous biology rather than a common mechanism for trigeminal neuralgia and SUNCT/SUNA. The treatment recommendations for SUNCT/SUNA, and our data, differ considerably to those for trigeminal neuralgia. Carbamazepine and oxcarbazepine are preferred treatments for trigeminal neuralgia, with lamotrigine and gabapentin having insufficient evidence for recommendation (20). In contrast, lamotrigine emerges as the most useful treatment for SUNCT, next topiramate, which now has randomised placebo-controlled trial data, while gabapentin emerges as the most useful treatment of SUNA. Remarkably, topiramate is not listed in the top tier treatments of trigeminal neuralgia (20). The finding of an abnormal vascular loop is common in classical trigeminal neuralgia (21,22). We report these loops in 10% of cases. An important limitation of our data is that they were not collected with this question in mind, scanners used were not uniform, and we only had clinically noted reporting. We remain impressed by the indifferent effects of surgery beyond what would be expected after manipulation in this region and anaesthetic. Lastly, functional imaging studies have established changes in the posterior hypothalamic region in SUNCT (23,24), whereas no such changes have been reported in trigeminal neuralgia. Taken together, the evidence to differentiate between SUNCT/SUNA and trigeminal neuralgia is strong. One possible issue is under-diagnosis of SUNA in what is, in our experience, often called atypical trigeminal neuralgia (25). This issue certainly deserves further attention.

A family history in SUNCT is reported in only one cohort (26). Regarding other trigeminal autonomic cephalalgias, there are reports of familial cluster headache (27–30), familial paroxysmal hemicrania (31) and familial hemicrania continua (32), as well as twins with cluster headache (33–35). Whether the rarity of familial cases reflects a non-genetic basis to the condition or simply the rarity of the problem remains an important unresolved issue.

The majority of SUNCT and SUNA cases were idiopathic in previous reports. SUNCT syndrome was delineated in patients with microprolactinomas (36) and macroprolactinomas (37,38), with attacks appearing on the same side as the tumors. A further five SUNCT cases were reported with pituitary adenomas, all with attacks ipsilateral to the side of the tumor (39); among the five patients, two of them did not improve after surgical removal of the tumor, one case was pain-free after surgery, one case was pain-free for 1 year then the pain relapsed with tumor recurrence, and one patient had a major reduction in headaches. In our cohort, there were four SUNCT and no SUNA cases with pituitary lesions (two macroadenomas and two microadenomas). When one considers TAC presentations and pituitary tumours (40) in the context of population-based pituitary tumour presentations (41), and the subsequent concordant course of the disorders, there does seem to be a relationship between TACs and acromegaly/prolactinoma not accounted for by chance.

In the literature, SUNCT patients were reported to be responsive to topiramate at doses of between 75 and 300 mg/day (42,43). The data from our reported cohort suggest about half of SUNCT patients find topiramate useful whereas few patients with SUNA find it useful. The open label SUNCT data are now supported by the placebo-controlled data that we report. SUNCT is rare, and doing large controlled trials in SUNCT prevention is challenging. The *N-of-1* crossover approach is appropriate for this patient group. The finding that two of the five patients clearly benefitted on the primary endpoint and one on an important secondary endpoint of reducing time spent in pain, supports the open label experience and offers a clear mandate to continue to offer topiramate to SUNCT patients.

The issue of inter-paroxysmal pain in SUNCT/SUNA, and more broadly in trigeminal autonomic cephalalgias, is an important pathophysiological issue. It can confuse clinicians and certainly can be an important part of the disability of these conditions. In these patients, a little more than half had some identified migrainous biology, which we defined operationally as a personal or family history of migraine. While this seems considerable, if one reviews the cumulative lifetime migraine incidence for episodic migraine, it is 43% in females (44). If one adds in patients with probable migraine and chronic migraine, it could be easily seen that the gene pool for migraine in females is about 50%. It could be argued that the rates we see here are a combination of that gene pool and the diagnostic bias of seeing a physician. Interestingly, inter-paroxysmal background pain was strongly associated with migrainous biology, as was a worsening of pain with movement, and the presence of photophobia and phonophobia. It may be argued that SUNCT and SUNA facilitate the expression of migrainousness, which expresses itself in these other features. Interestingly, nausea did not seem to be associated, which may be because it is relatively less common or because the SUNCT/SUNA effect on migraine pathways is not universal.

In summary, a very substantial cohort of patients with SUNCT and SUNA is presented. Based on the phenotypic presentation of the pain features and the distribution of cranial autonomic symptoms, the two seem different. This is supported by differences in response to preventive therapies, particularly topiramate, for which we supply data from a randomised placebo-controlled trial. An analysis of the clinical, therapeutic, and pathophysiological findings in SUNCT/SUNA supports their differentiation from classical trigeminal neuralgia. The findings of associations between background migrainous biology and features such as inter-paroxysmal pain and aggravation of pain with movement, suggest SUNCT and SUNA can trigger the expression of an underlying migrainous process that colours the clinical presentation. SUNCT and SUNA seem well placed as trigeminal autonomic cephalalgias (TACs), and our clinical data offer both directions for new work and consolidate what is known about these rare and highly disabling conditions.

## Clinical implications

- Short-lasting unilateral neuralgiform headache attacks with conjunctival injection and tearing (SUNCT) and short-lasting unilateral neuralgiform headache attacks with cranial autonomic symptoms (SUNA) are two rare headache syndromes classified broadly as trigeminal autonomic cephalalgias (TACs).
- Regarding cranial autonomic features, SUNA patients were most likely to have lacrimation and ptosis.
- Patients with SUNCT and SUNA who had had a personal or family history of migraine were more likely to have photophobia, phonophobia and persistent pain between attacks.
- A randomized placebo-controlled cross-over trial of topiramate in SUNCT using an *N-of-1* design supports offering this therapy to patients with SUNCT.
